# Interrelationship of LV mass, focal fibrosis by LGE, and diffuse fibrosis by T1-changes in patients with hypertrophic cardiomyopathy

**DOI:** 10.1186/1532-429X-14-S1-P166

**Published:** 2012-02-01

**Authors:** Rahul Potluri, Christopher A Miller, Matthias Schmitt

**Affiliations:** 1 Department of Cardiac MR, University Hospital of South Manchester, Manchester, UK

## Background

Hypertrophic cardiomyopathy (HCM) is characterised by left ventricular hypertrophy, myofibre disarray as well as interstitial and replacement fibrosis. These characteristics are likely the underlying cause of reduced ventricular compliance and cardiac arrhythmias which subsequently impact on quality of life and outcome.

Cardiac magnetic resonance (CMR) based late gadolinium imaging (LGE) has emerged as a prognosticator in multiple cardiac myopathies, including HCM, and is able to demonstrate a type of myocardial scarring that has been likened to replacement fibrosis. CMR based T1 mapping techniques on the other hand are thought to be able to also detect a more diffuse increase in collagen volume fraction of myocardial tissue (i.e. diffuse fibrosis). It has been argued that the latter type of fibrosis may be reversible up to a certain stage. However, even in the physiologically unlikely scenario that both fibrotic processes were independent of each other above mentioned CMR based technologies are not.

## Aims

We therefore sought to investigate the relationship between LV mass (LVM), LGE imaging, using multiple cut-off points for signal differences to normal myocardium, and T1 mapping in order to gain a better understanding of myocardial tissue characteristics (i.e scar volume/diffuse fibrosis) as well as the interconnection of LVM, LGE and T1 mapping as currently applied.

## Methods

Twenty-one consecutive HCM patients attending for clinically indicated scans (1.5T Avanto, 32 channel phased array cardiac-coil, Siemens, Germany) underwent T1 mapping pre- and 10 minutes post gadolinium contrast using a modified look locker inversion recovery (MOLLI) sequence (analysed using the T1 mapping plugin (Emerix) for Osirix (v3.9.1, Pixmeo). LGE images were acquired immediately after post-contrast T1 mapping using an inversion recovery gradient echo sequence and analysed with thresholds of 2 and 6 standard deviations (SD) and the full width half maximum (FWHM) method (Qmass, v7.2, Leiden). Statistical analysis was performed using SPSS version 19.0.

## Results

**Figure 1:**
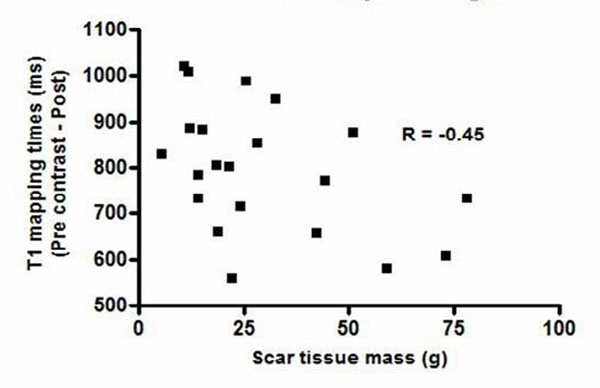
Association between T1 mapping and scar tissue analyses using LGE.

Fourteen patients were male, mean age 54±16 years. Mean left ventricular mass (LVM) was 163.4±57.0g. Mean focal fibrosis masses (MFF) were 29.5±20.7g using FWHM, 65.7±32.1g using 2 SD threshold and 19.6±15.5g using 6 SD threshold. Mean pre-contrast T1 mapping time was 1217±110ms compared with post-contrast of 421±73.8ms. Mean difference between pre and post T1 mapping times (T1Diff) was 797±137sms. Age did not show a significant relationship with LVM, MFF or T1Diff. There was a significant correlation between LVM and MFF (r=0.67;p=0.001 using the FWHM and 2SD thresholds). T1Diff was inversely correlated with MFF using FWHM threshold(p=0.043, Figure 1) and with LVM (r=-0.43;p=0.047).

## Conclusions

In HCM, T1 Diff is inversely correlated to FWHM derived MFF and LVM. LVM and MFF (using FWHM) are also inversely correlated.

## Funding

Nothing to disclose.

